# Supplementary material for: MRI-based microthrombi detection in stroke with polydopamine iron oxide
Source: Nat Commun. 2024 Jun 13;15:5070. doi: 10.1038/s41467-024-49480-x (PMC11176332; doi:10.1038/s41467-024-49480-x)
Supplement: Supplementary file 1 — Supplementary information file. [file 41467_2024_49480_MOESM1_ESM.pdf]

Supplementary Information

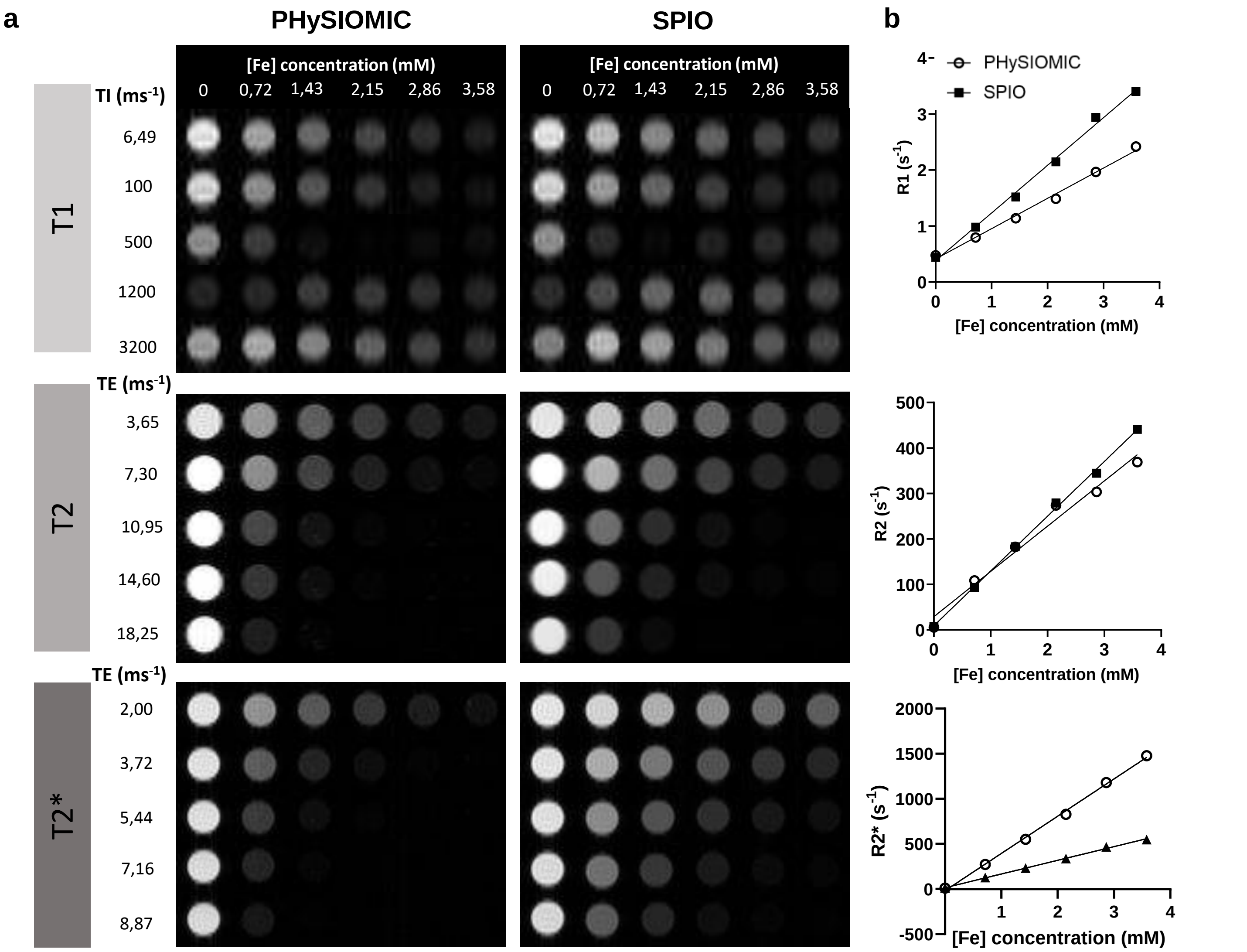

Supplementary Figure 1. PHySIOMIC and SPIO relaxivities on T1 , T2 and T2 *-weighted MRI. a, MR images of PHySIOMIC and SPIO particles at increasing concentrations embedded in an agarose gel (2%), and at various inversion times (TI) for T1 -weighted imaging, as well as different echo times (TE) for T2 -weighted and T2 *-weighted images. **b,** Corresponding relaxivity values (R1, R2, and R2*) as a function of PHYSIOMIC and SPIO concentrations, demonstrating lower R1 relaxivity and nearly 2 times higher R2* relaxivity for PHYSIOMIC compared to SPIO (n=1 particles preparation). Source data are provided as a Source Data file.

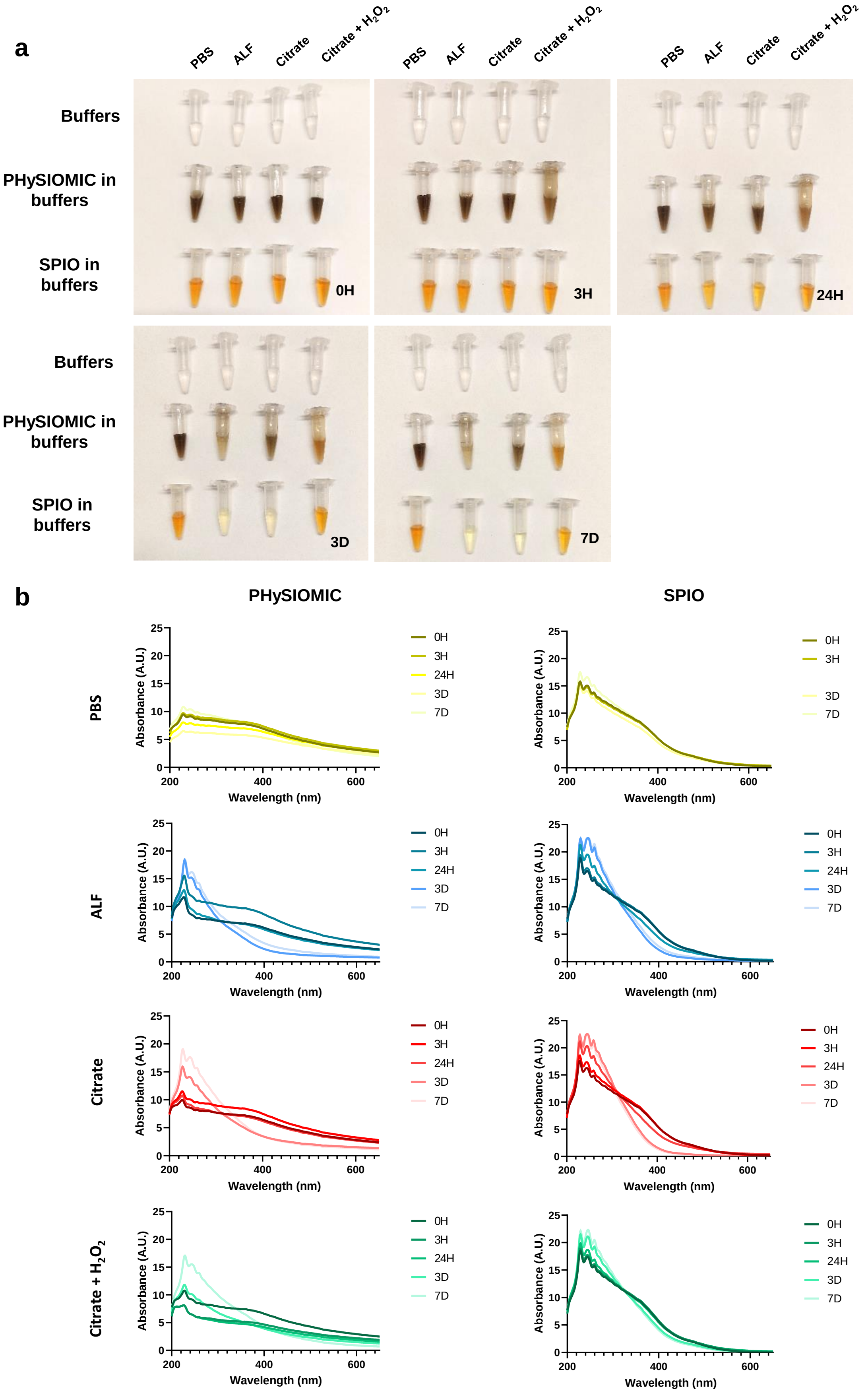

Supplementary Figure 2. In vitro degradation of PHySIOMIC and SPIO. a, Images illustrating the in vitro degradation of PHySIOMIC and SPIO particles at equal concentrations in Phosphate Buffered Saline (PBS), Artificial Lysosomal Fluid (ALF), Citrate, and Citrate with hydrogen peroxide (H2O2) buffers over a 7-day period. **b,** The corresponding absorbance values measured at each time point across the wavelength spectrum of PHySIOMIC and SPIO in the aforementioned buffers using UV-Vis spectroscopy (n=1 particles preparation). Source data are provided as a Source Data file.

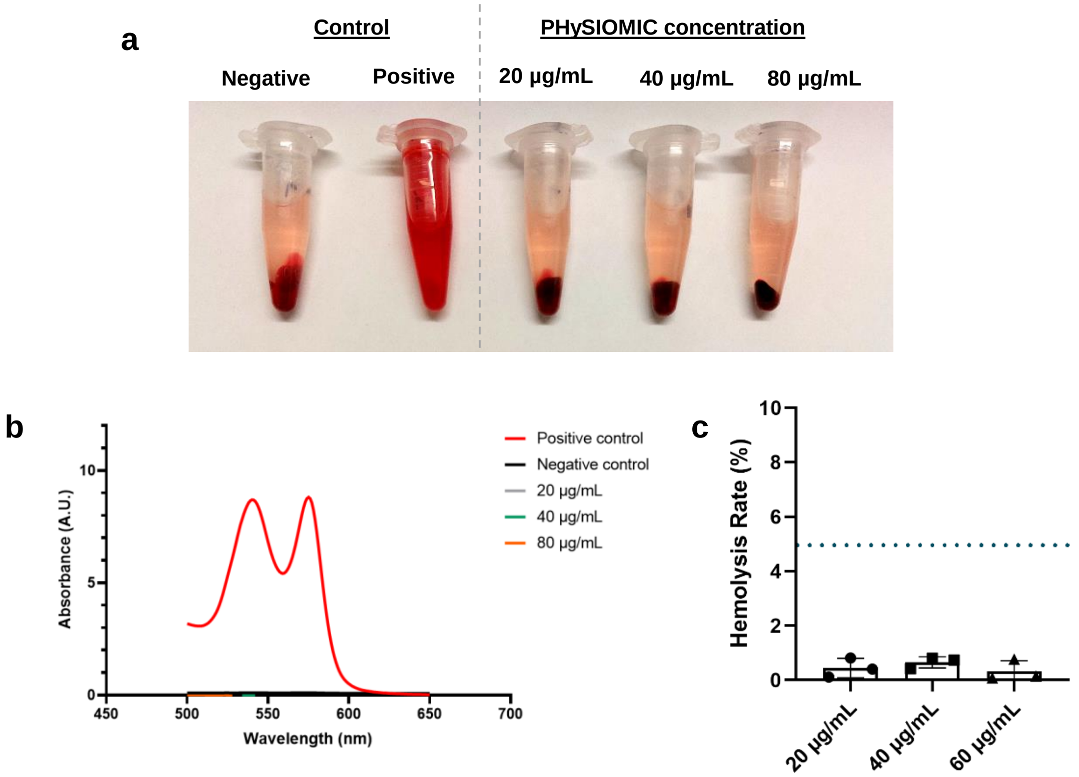

Supplementary Figure 3. Evaluation of hemolysis effect of PHySIOMIC. a, Images showing human erythrocytes in physiological serum with the addition of increasing concentrations of PHySIOMIC particles (20, 40, and 80 µg.mL^{-1}), as well as the addition of distilled water (positive control) or PBS buffer (negative control), after centrifugation. **b,** Absorbance measurements of the supernatant in each test tube across the wavelength spectrum. **c,** Corresponding hemolysis rates for each concentration of PHySIOMIC. Hemolysis rates exceeding 5% (indicated by the dotted line) are considered detrimental (n=3 tubes per group, One-way ANOVA). Results are presented as mean ± SD . Source data are provided as a Source Data file.

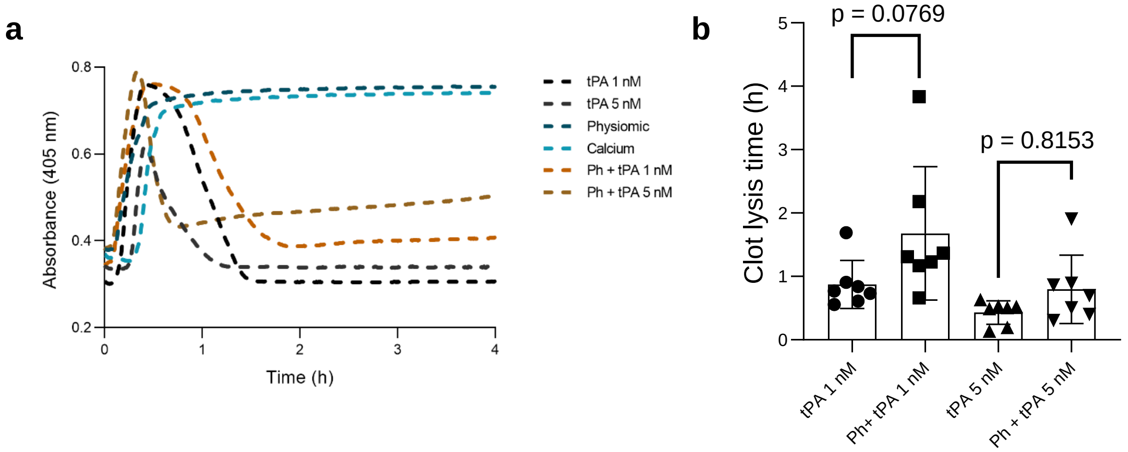

Supplementary Figure 4. Influence of PHySIOMIC particles on in vitro clot lysis time. a, Absorbance measurements over time in wells containing plasma from healthy donors. Clot formation is initiated by adding calcium to the wells at the start. The decrease in absorbance reflects clot lysis. Curves are displayed for clots exposed to 1nM and 5nM tPA, calcium alone (positive control), PHySIOMIC alone, and PHySIOMIC + 1nM and 5nM tPA. **b,** Clot lysis times are measured for each group. Results, presented as mean ± SD, were not statistically significant for each tPA concentration (n = 7 tube samples, Friedman test with Dunn's multiple comparisons test). Source data are provided as a Source Data file.

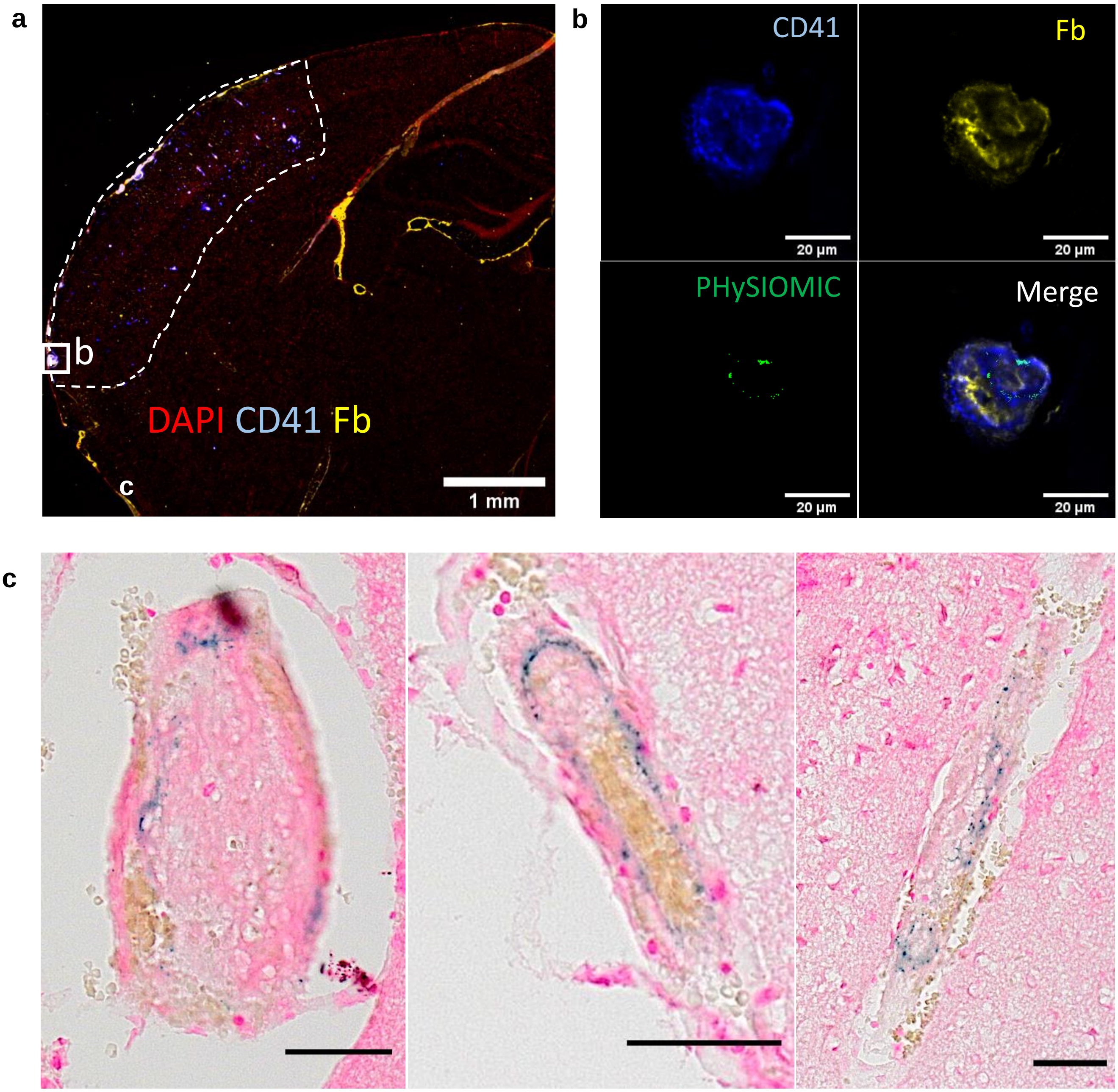

Supplementary Figure 5. Microthrombi presence in the ipsilateral cortex after thrombin injection into the middle cerebral artery, highlighted by immunohistochemistry. a, Microscope image of the ipsilateral cortex of the mouse after thrombin injection. Distribution of the proximal clot and microthrombi revealed by combination of platelet (CD41) and fibrin (Fb) fluorescence, with cell nuclei staining (DAPI) to observe brain structures. **b**, Microthrombi and PHySIOMIC observation in confocal microscopy. PHySIOMIC were visualized via laser reflexion from their polydopamine content (here in green). **c.** Perls' staining was performed on brain tissue collected after PHySIOMIC injection to visualize the presence of PHySIOMIC in the brain. The staining revealed the ferric iron (in blue) present in PHySIOMIC, and the tissue was counterstained with nuclear fast red for observation. Optical microscopy confirmed the presence of PHySIOMIC surrounding thrombi and microthrombi in the ipsilateral cortex, scale bar = 50 µm (Observations on n=5 animals). Source data are provided as a Source Data file.

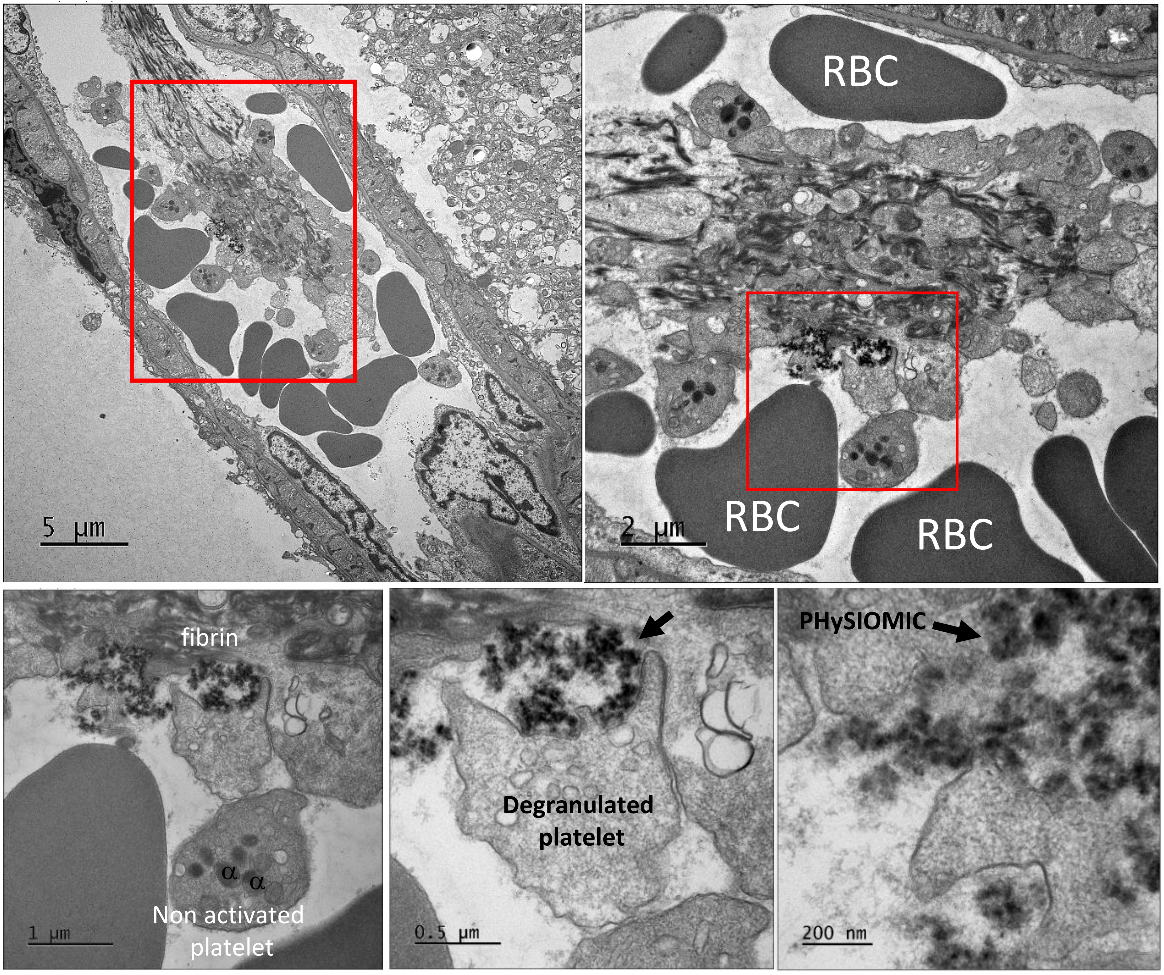

Supplementary Figure 6. TEM was performed on brain tissue collected after PHySIOMIC injection to visualize the presence of PHySIOMIC in the brain. The images show PHySIOMIC particles (black arrow) positioned next to degranulated platelets. RBC: Red Blood Cell. (Observation on 1 animal). Source data are provided as a Source Data file.

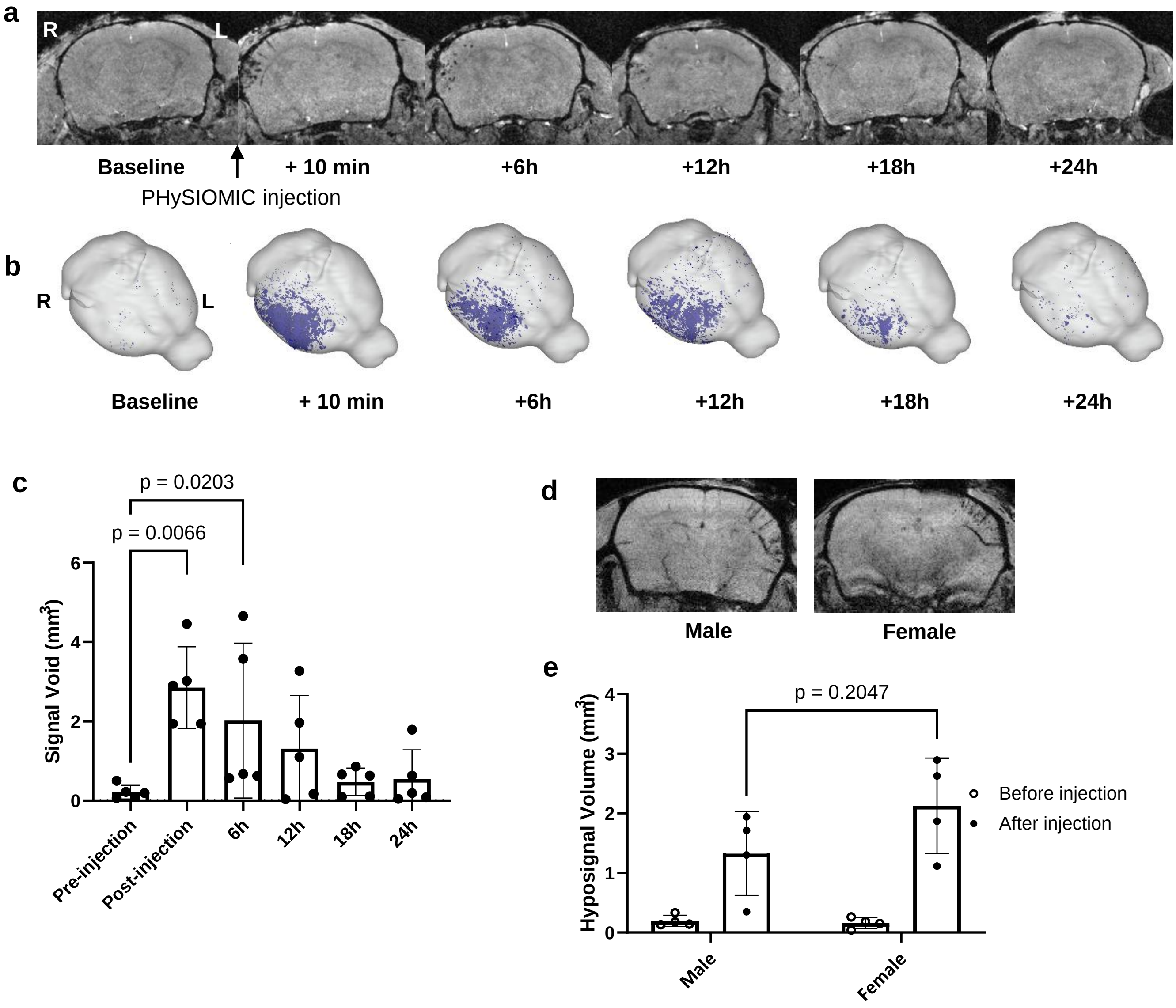

Supplementary Figure 7. PHySIOMIC' signal decreases on MRI monitoring for 24 Hours following Injection and is similar between male and female. a, T_2*-weighted images acquired before (baseline), 10 minutes after injection, and then at 6 hours, 12 hours, 18 hours, and 24 hours post-injection, illustrating the reduction in hypointensity induced by the particles. **b,** A 3D representation of the particle-induced signal (in blue) present in the cerebral cortex. **c,** Quantification of the signal volume in mm³ calculated at each time point post-injection (n=5 animals, Friedman test with Dunn's multiple comparisons). Data are presented as mean ± SD. **d,** T_2*-weighted images acquired directly after PHySIOMIC injection in male and female mice. **e,** The hyposignal that appears with PHySIOMIC is similar in both groups, and no significant difference is observed between male and female groups with hyposignal volume quantification (n=4 animals, Two-way ANOVA with Sidak's multiple comparisons test). R and L indicate right and left side of the presented brain. Source data are provided as a Source Data file.

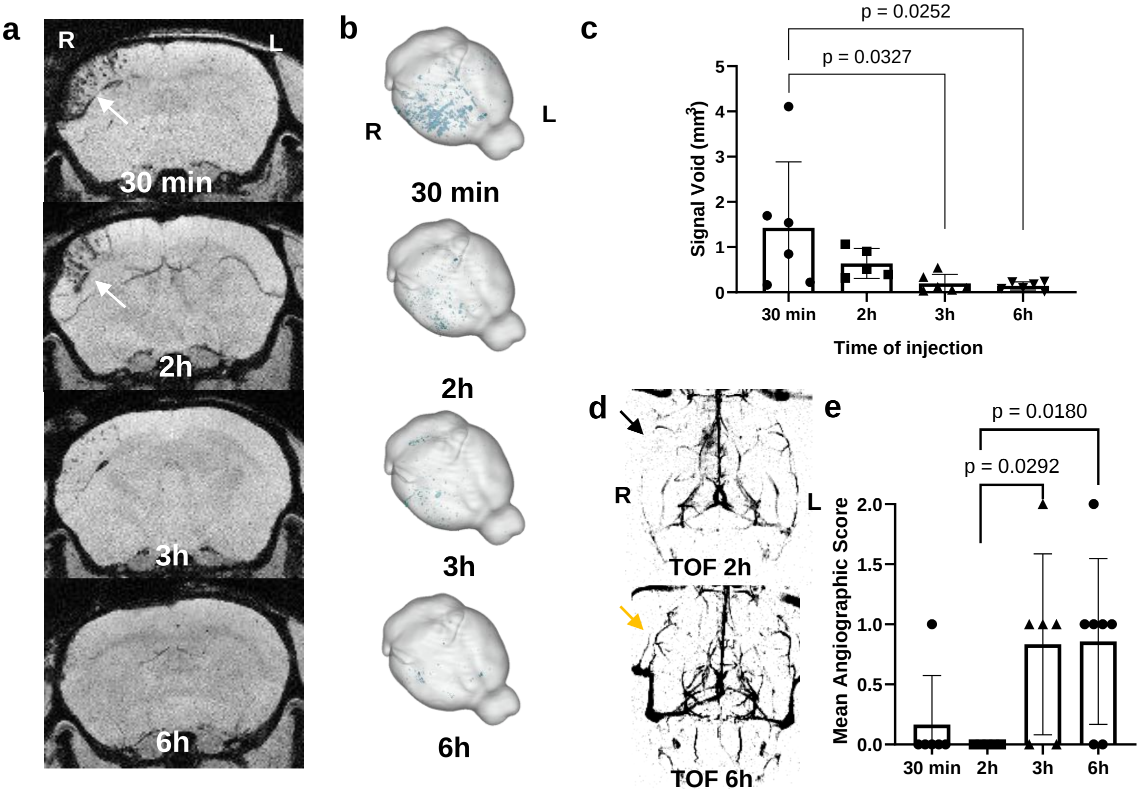

Supplementary Figure 8. Reduction in microthrombosis observed by injecting PHySIOMIC at various time points after MCAO, corresponding to spontaneous recanalization induced by the model. a, T2*-weighted MRI images acquired showing the PHySIOMIC signal in the ischemic area (indicated by the yellow dotted line) after injection at 30 minutes, 2 hours, 3 hours, or 6 hours post-occlusion. **b,** A 3D representation of the hypointensity induced by PHySIOMIC (in blue) in the cerebral cortex of mice. **c,** Quantification of the signal void in mm³ at each post-occlusion time. A statistical difference is observed at 3 and 6 hours compared to 30 minutes post-occlusion (n=5 animals, One-Way ANOVA with Dunnett's multiple comparisons test). Results as mean ± SD. **d,** Spontaneous recanalization observed in this MCAO model through angiographic images (TOF, time-of-flight), with no recanalization at 2 hours (indicated by the black arrow) but initiating recanalization at 6 hours (indicated by the yellow arrow). **e,** A graph of angiographic scores assigned, based on TOF images, at each post-occlusion time (0 = No recanalization, 1 = Partial recanalization, 2 = Total recanalization) (n=5-7 animal, Kruskal-Wallis with uncorrected Dunnett's multiple comparisons test s). Results as mean ± SD. Source data are provided as a Source Data file.

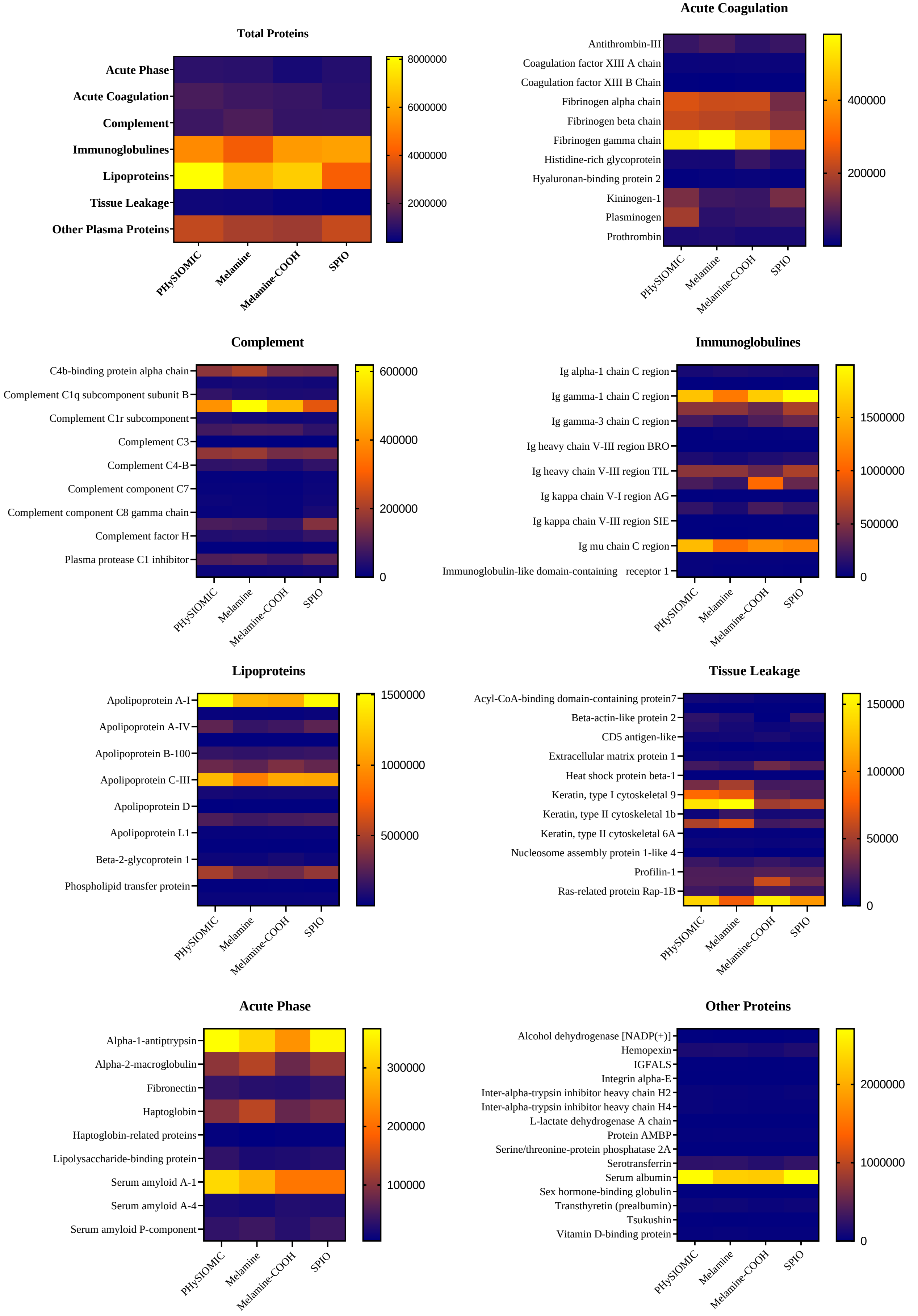

Supplementary Figure 9. Composition of the protein corona (PC) of PHySIOMIC, Melamine-amine, and Melamine-carboxylic particles in human plasma evaluated through proteomics. Measurement of mass spectrometric signal intensity, revealing the relative proportions of proteins within the PC, classified by their biological functions, following a 1-hour incubation of particles in human plasma and detailed information on the major proteins involved in each class within the protein corona composition PHySIOMIC, Melamine and Melamine-COOH particles analyzed in triplicate (n=1 sample preparation) and Detailed information on the major proteins involved in each class within the protein corona composition for each type of particle. Source data are provided as a Source Data file.

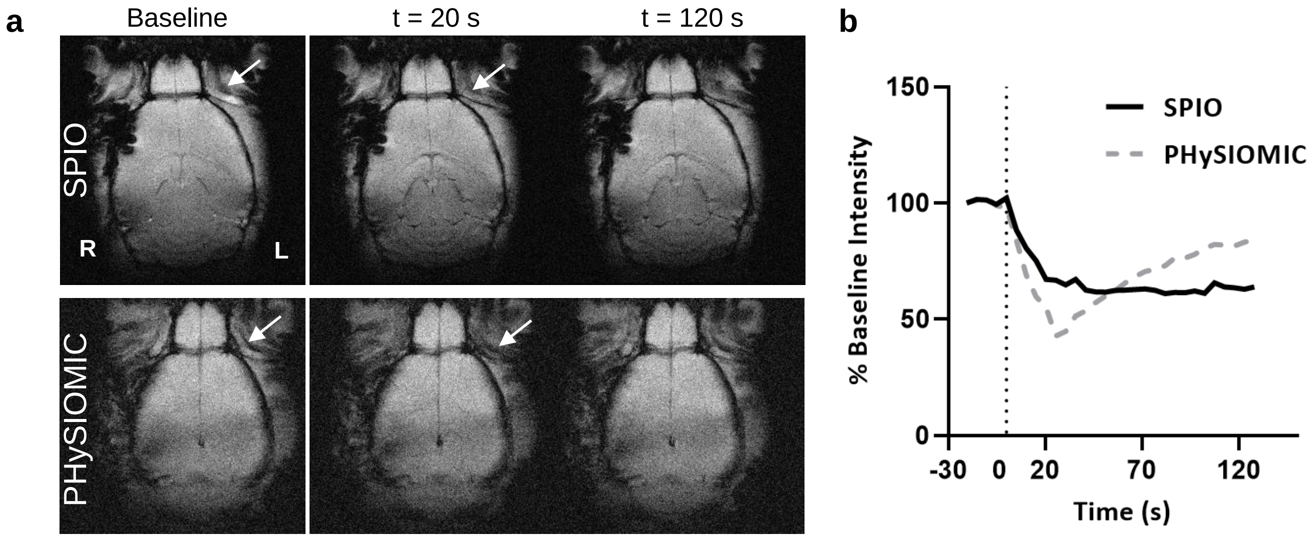

Supplementary Figure 10. Estimation of the half-life of PHySIOMIC in the bloodstream compared to that of SPIO. a, Dynamic ultrafast low resolution T2*-weighted coronal acquisition were performed to monitor the particle in circulation and accumulation. Images are shown at baseline and after the injection of particles. The particles are visible at the retro-orbital vein (indicated by the white arrow). Video of the acquisition (Supplementary movie 1) with PHySIOMIC injection is provided with image substraction that features the PHySIOMIC seen in the retro-orbital vein, followed by the accumulation at the microthrombi area. **b,** Measurement of signal intensity relative to the baseline signal at the retro-orbital vein over time (n = 1 animal). Source data are provided as a Source Data file.

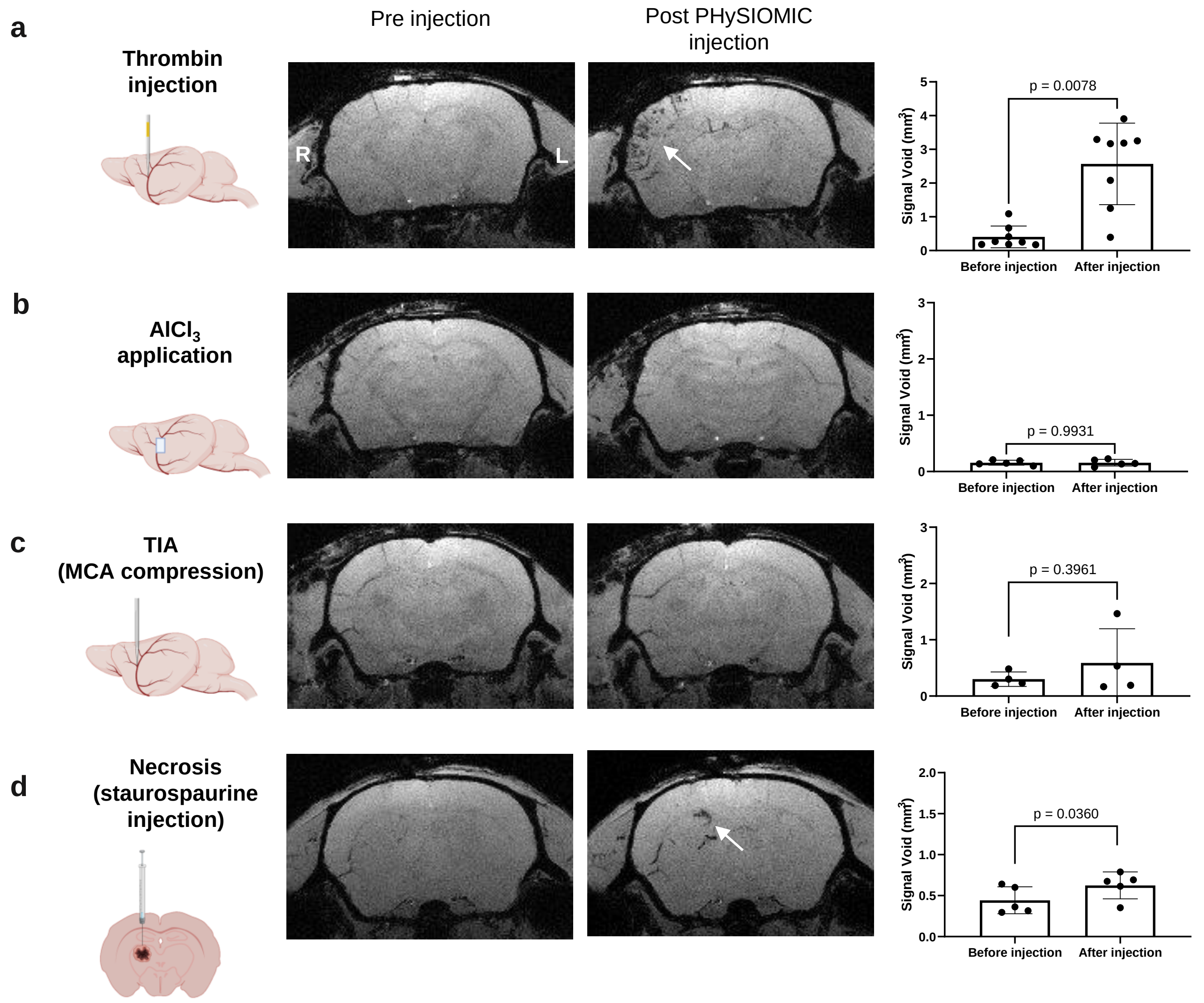

Supplementary Figure 11. Microthrombi detection in different stroke models and necrosis after PHySIOMIC injection. a,b, PHySIOMIC indicates the presence of microthrombi in the cerebral cortex of the MCAO model induced by thrombin injection (n=8 animals, Wilcoxon matched-pairs signed rank test, two-tailed) but not in the model induced by aluminum chloride application (AlCl3) (n=5 animals, paired t-test, two-tailed). Results presented as mean ± SD. **c,** No hypointensity of microthrombi is observed in a TIA model induced by ACA compression (n=4 animals, paired t-test, two-tailed). **d,** Microthrombi surrounding necrosis is evident in the model induced by staurosporine injection in the striatum (n=5 animals, paired t-test, two-tailed). Source data are provided as a Source Data file.
