# Supplementary material for: MRI-based microthrombi detection in stroke with polydopamine iron oxide
Source: Nat Commun. 2024 Jun 13;15:5070. doi: 10.1038/s41467-024-49480-x (PMC11176332; doi:10.1038/s41467-024-49480-x)
Supplement: Supplementary file 3 — Description of Additional Supplementary Files [file 41467_2024_49480_MOESM3_ESM.docx]

MRI-Based Microthrombi Detection in Stroke with Polydopamine Iron Oxide

**Author list**

Charlène Jacqmarcq^1^, Audrey Picot^1^, Jules Flon^1^, Florent Lebrun^1^, Sara Martinez de Lizarrondo^1^, Mikaël Naveau^2^, Benoît Bernay^3^, Didier Goux^4^, Marina Rubio^1^, Aurélie Malzert-Fréon^5^, Anita Michel^6^, Fabienne Proamer^6^, Pierre Mangin^6^, Maxime Gauberti^1,7^, Denis Vivien^1,8*^, Thomas Bonnard^1*^

**Description of Additional Supplementary files**

**File Name: Supplementary Movie 1**

**Description:**

The bottom part show the dynamic ultrafast low resolution T2*-weighted coronal acquisition performed to monitor the particle in circulation and accumulation with PHySIOMIC injection. The upper part show the corresponding substraction featuring the PHySIOMIC seen in the retro-orbital vein, followed by the accumulation at the microthrombi area. The time in second post PHySIOMIC injection is indicated for each images.
