# Supplementary figures and images for: MRI-based microthrombi detection in stroke with polydopamine iron oxide
Source: Nat Commun. 2024 Jun 13;15:5070. doi: 10.1038/s41467-024-49480-x (PMC11176332; doi:10.1038/s41467-024-49480-x)

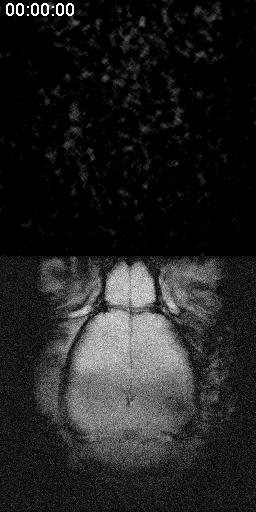

Supplement: Supplementary file 4 — Supplementary Movie 1 [file 41467_2024_49480_MOESM4_ESM.gif]
