# Supplementary material for: MRI-based microthrombi detection in stroke with polydopamine iron oxide
Source: Nat Commun. 2024 Jun 13;15:5070. doi: 10.1038/s41467-024-49480-x (PMC11176332; doi:10.1038/s41467-024-49480-x)
Supplement: Supplementary file 5 — Reporting Summary [file 41467_2024_49480_MOESM5_ESM.pdf]

nature portfolio

Corresponding author(s): Thomas Bonnard, Denis Vivien

Last updated by author(s): May 22, 2024

Reporting Summary

Nature Portfolio wishes to improve the reproducibility of the work that we publish. This form provides structure for consistency and transparency in reporting. For further information on Nature Portfolio policies, see our Editorial Policies and the Editorial Policy Checklist.

Statistics

For all statistical analyses, confirm that the following items are present in the figure legend, table legend, main text, or Methods section.

n/a	Confirmed	
☐	☒	The exact sample size (*n*) for each experimental group/condition, given as a discrete number and unit of measurement
☐	☒	A statement on whether measurements were taken from distinct samples or whether the same sample was measured repeatedly
☐	☒	The statistical test(s) used AND whether they are one- or two-sided<br>*Only common tests should be described solely by name; describe more complex techniques in the Methods section.*
☐	☒	A description of all covariates tested
☐	☒	A description of any assumptions or corrections, such as tests of normality and adjustment for multiple comparisons
☐	☒	A full description of the statistical parameters including central tendency (e.g. means) or other basic estimates (e.g. regression coefficient) AND variation (e.g. standard deviation) or associated estimates of uncertainty (e.g. confidence intervals)
☐	☒	For null hypothesis testing, the test statistic (e.g. *F*, *t*, *r*) with confidence intervals, effect sizes, degrees of freedom and *P* value noted<br>*Give P values as exact values whenever suitable.*
☒	☐	For Bayesian analysis, information on the choice of priors and Markov chain Monte Carlo settings
☒	☐	For hierarchical and complex designs, identification of the appropriate level for tests and full reporting of outcomes
☒	☐	Estimates of effect sizes (e.g. Cohen's *d*, Pearson's *r*), indicating how they were calculated

Our web collection on statistics for biologists contains articles on many of the points above.

Software and code

Policy information about availability of computer code

Data collection	MRI Images : Paravision (Bruker, v.6.0.1),<br>Two-photon images : PrairieView (Bruker, v.5.7),<br>Confocal Images : Leica Application Suite AF (LAS AF v.3);<br>Virtual Scanner : Olympus CellSens
Data analysis	Data statistics : GraphPad (v.8.0);<br>Histology images : QuPath (v0.5.1) and ImageJ (v.1.52i) ,<br>MRI images : Slicer (v.4.11) and ImageJ (v.1.51i)

For manuscripts utilizing custom algorithms or software that are central to the research but not yet described in published literature, software must be made available to editors and reviewers. We strongly encourage code deposition in a community repository (e.g. GitHub). See the Nature Portfolio guidelines for submitting code & software for further information.

Data

Policy information about availability of data

All manuscripts must include a data availability statement. This statement should provide the following information, where applicable:

- Accession codes, unique identifiers, or web links for publicly available datasets
- A description of any restrictions on data availability
- For clinical datasets or third party data, please ensure that the statement adheres to our policy

The data supporting the findings from this study are available within the manuscript and its supplementary information. Source data are provided with this paper. Proteomics data have been deposited in iProX under accession code IPX0008212001 https://www.iprox.cn//page/. All magnetic resonance imaging and confocal files are not provided as the size is too important but any raw data will be available from the corresponding author upon reasonable request.

Research involving human participants, their data, or biological material

Policy information about studies with human participants or human data. See also policy information about sex, gender (identity/presentation), and sexual orientation and race, ethnicity and racism.

Reporting on sex and gender	In our study, we used blood samples from both sex.
Reporting on race, ethnicity, or other socially relevant groupings	Data on the race, ethnicity, or social group of the donors who participated were not collected.
Population characteristics	The blood was collected from healthy adult donors who voluntarily presented themselves at the EFS for a blood donation and had received approval from a doctor for the donation.
Recruitment	Healthy volunteers were recruited by the EFS among the donor for blood bank. Donors were asked if they agree to give a supplemental sample of blood for research purpose. They signed a consent form and gave 3 mL blood samples collected on citrate.
Ethics oversight	Convention de cession de produits issus du sang ou de ses composants à but non thérapeutiques n° PLER /2021/005 established between the EFS Hauts-de-France – Normandie and the Institut National de la Santé et de la Recherche Médical (INSERM)

Note that full information on the approval of the study protocol must also be provided in the manuscript.

Field-specific reporting

Please select the one below that is the best fit for your research. If you are not sure, read the appropriate sections before making your selection.

☒ Life sciences ☐ Behavioural & social sciences ☐ Ecological, evolutionary & environmental sciences

For a reference copy of the document with all sections, see nature.com/documents/nr-reporting-summary-flat.pdf

Life sciences study design

All studies must disclose on these points even when the disclosure is negative.

Sample size	The sample sizes were determined based on previous experiments for the in vivo part. Papers from the laboratory already published show that 5 animals may be sufficient to demonstrate a discriminating signal in MRI. For the microfluidics, an initial experiment is conducted with n=3, then we use a power calculation (https://clincalc.com/stats/samplesize.aspx).
Data exclusions	Some data were excluded from the study due to non-compliance with the pre-established criteria outlined in the Materials and Methods section (Exclusion criteria).
Replication	We verified the reproducibility of the results by measuring the samples in triplicate for proteomic analysis, as well as the measurement of particle relaxivities in MRI, the study of particle biodergradation in solution, and hemolysis. Additionally, we measured the adsorption of fibrinogen to the particle surface by taking five measurements due to slight variability in the results. For all other experiments, any experiments that were not exluded based on exlusion criteria were included in the analysis.
Randomization	The groups were assigned to the mice in a way to avoid biases due to belonging to the same cage or biases due to different experimentation days.
Blinding	Investigators were blinded to the experimental groups during the data analysis but not during data collection as either the design of experiments do not allow it (comparison of different time points) either the control conditions imply the use of a different tube, making blinding of the investigator not possible.

Reporting for specific materials, systems and methods

We require information from authors about some types of materials, experimental systems and methods used in many studies. Here, indicate whether each material, system or method listed is relevant to your study. If you are not sure if a list item applies to your research, read the appropriate section before selecting a response.

Materials & experimental systems

n/a	Involved in the study	
☐	☒	Antibodies
☐	☐	Eukaryotic cell lines
☐	☐	Palaeontology and archaeology
☐	☒	Animals and other organisms
☐	☐	Clinical data
☐	☐	Dual use research of concern
☐	☐	Plants

Methods

n/a	Involved in the study	
☐	☐	ChIP-seq
☐	☐	Flow cytometry
☐	☒	MRI-based neuroimaging

Antibodies

Antibodies used	Here are the antibodies used (see also Materials and Methods scetion) : anti-fibrinogen/fibrin (1:1000; sheep antiserum againt fibrinogen prepared at the National institute for agronomic research, France), platelet-specific marker anti-CD41 (1:1000; BD Biosciences). Primary antibodies were revealed using Fab'2 fragments of donkey anti-rat, goat, sheep IgG linked to FITC, TRITC or DyLight 629 (1:800, Jackson ImmunoResearch, USA).
Validation	The specificity of immunostainings was checked during each immunohistochemistry experiment by the absence of staining when primary antibodies were omitted.<br>Both primary antibodies were used in previous published papers (Le Behot et al., 2014 Blood. doi : 10.1182/blood-2013-12-543074; Martinez de Lizarrondo et al., 2017. Circulation. doi : 10.1161/CIRCULATIONAHA.117.027290).

Eukaryotic cell lines

Policy information about cell lines and Sex and Gender in Research

Cell line source(s)	*State the source of each cell line used and the sex of all primary cell lines and cells derived from human participants or vertebrate models.*
Authentication	*Describe the authentication procedures for each cell line used OR declare that none of the cell lines used were authenticated.*
Mycoplasma contamination	*Confirm that all cell lines tested negative for mycoplasma contamination OR describe the results of the testing for mycoplasma contamination OR declare that the cell lines were not tested for mycoplasma contamination.*
Commonly misidentified lines (See ICLAC register)	*Name any commonly misidentified cell lines used in the study and provide a rationale for their use.*

Palaeontology and Archaeology

Specimen provenance	*Provide provenance information for specimens and describe permits that were obtained for the work (including the name of the issuing authority, the date of issue, and any identifying information). Permits should encompass collection and, where applicable, export.*
Specimen deposition	*Indicate where the specimens have been deposited to permit free access by other researchers.*
Dating methods	*If new dates are provided, describe how they were obtained (e.g. collection, storage, sample pretreatment and measurement), where they were obtained (i.e. lab name), the calibration program and the protocol for quality assurance OR state that no new dates are provided.*

☐ Tick this box to confirm that the raw and calibrated dates are available in the paper or in Supplementary Information.

Ethics oversight	*Identify the organization(s) that approved or provided guidance on the study protocol, OR state that no ethical approval or guidance was required and explain why not.*

Note that full information on the approval of the study protocol must also be provided in the manuscript.

Animals and other research organisms

Policy information about studies involving animals; ARRIVE guidelines recommended for reporting animal research, and Sex and Gender in Research

Laboratory animals	All studies were conducted on male or female Swiss mice (age 8-10 weeks; weight 35-45g ; CURB, Caen, France). Mice were housed in a temperature-controlled room (21°C), hygrometry (55%), brightness (100 lux), on a 12-hour light/12-hour dark cycle with food and water ad libitum. They were ccommodated in small stable social groups (3 to 5 per cage) with a minimum enrichment (a cardboard roll + a sheet of paper towel), and handled only by experienced staff.
Wild animals	The study did not involve wild animals.
Reporting on sex	Sex was considered in the study design and we demonstrated no differences between male and female regarding microthrombi presence and targeting.
Field-collected samples	The study did not involve samples collected from the field.
Ethics oversight	This study was approved by the local ethical committee of Normandy (CENOMEXA).

Note that full information on the approval of the study protocol must also be provided in the manuscript.

Clinical data

Policy information about clinical studies

All manuscripts should comply with the ICMJEguidelines for publication of clinical research and a completedCONSORT checklist must be included with all submissions.

Clinical trial registration	*Provide the trial registration number from ClinicalTrials.gov or an equivalent agency.*
Study protocol	*Note where the full trial protocol can be accessed OR if not available, explain why.*
Data collection	*Describe the settings and locales of data collection, noting the time periods of recruitment and data collection.*
Outcomes	*Describe how you pre-defined primary and secondary outcome measures and how you assessed these measures.*

Dual use research of concern

Policy information about dual use research of concern

Hazards

Could the accidental, deliberate or reckless misuse of agents or technologies generated in the work, or the application of information presented in the manuscript, pose a threat to:

No	Yes	
☐	☐	Public health
☐	☐	National security
☐	☐	Crops and/or livestock
☐	☐	Ecosystems
☐	☐	Any other significant area

Experiments of concern

Does the work involve any of these experiments of concern:

No	Yes	
☐	☐	Demonstrate how to render a vaccine ineffective
☐	☐	Confer resistance to therapeutically useful antibiotics or antiviral agents
☐	☐	Enhance the virulence of a pathogen or render a nonpathogen virulent
☐	☐	Increase transmissibility of a pathogen
☐	☐	Alter the host range of a pathogen
☐	☐	Enable evasion of diagnostic/detection modalities
☐	☐	Enable the weaponization of a biological agent or toxin
☐	☐	Any other potentially harmful combination of experiments and agents

Plants

Seed stocks	*Report on the source of all seed stocks or other plant material used. If applicable, state the seed stock centre and catalogue number. If plant specimens were collected from the field, describe the collection location, date and sampling procedures.*
Novel plant genotypes	*Describe the methods by which all novel plant genotypes were produced. This includes those generated by transgenic approaches, gene editing, chemical/radiation-based mutagenesis and hybridization. For transgenic lines, describe the transformation method, the number of independent lines analyzed and the generation upon which experiments were performed. For gene-edited lines, describe the editor used, the endogenous sequence targeted for editing, the targeting guide RNA sequence (if applicable) and how the editor was applied.*
Authentication	*Describe any authentication procedures for each seed stock used or novel genotype generated. Describe any experiments used to assess the effect of a mutation and, where applicable, how potential secondary effects (e.g. second site T-DNA insertions, mosiacism, off-target gene editing) were examined.*

ChIP-seq

Data deposition

☐ Confirm that both raw and final processed data have been deposited in a public database such as GEO.

☐ Confirm that you have deposited or provided access to graph files (e.g. BED files) for the called peaks.

Data access links<br>*May remain private before publication.*	*For "Initial submission" or "Revised version" documents, provide reviewer access links. For your "Final submission" document, provide a link to the deposited data.*
Files in database submission	*Provide a list of all files available in the database submission.*
Genome browser session<br>(e.g. UCSC)	*Provide a link to an anonymized genome browser session for "Initial submission" and "Revised version" documents only, to enable peer review. Write "no longer applicable" for "Final submission" documents.*

Methodology

Replicates	*Describe the experimental replicates, specifying number, type and replicate agreement.*
Sequencing depth	*Describe the sequencing depth for each experiment, providing the total number of reads, uniquely mapped reads, length of reads and whether they were paired- or single-end.*
Antibodies	*Describe the antibodies used for the ChIP-seq experiments; as applicable, provide supplier name, catalog number, clone name, and lot number.*
Peak calling parameters	*Specify the command line program and parameters used for read mapping and peak calling, including the ChIP, control and index files used.*
Data quality	*Describe the methods used to ensure data quality in full detail, including how many peaks are at FDR 5% and above 5-fold enrichment.*
Software	*Describe the software used to collect and analyze the ChIP-seq data. For custom code that has been deposited into a community repository, provide accession details.*

Flow Cytometry

Plots

Confirm that:

☐ The axis labels state the marker and fluorochrome used (e.g. CD4-FITC).

☐ The axis scales are clearly visible. Include numbers along axes only for bottom left plot of group (a 'group' is an analysis of identical markers).

☐ All plots are contour plots with outliers or pseudocolor plots.

☐ A numerical value for number of cells or percentage (with statistics) is provided.

Methodology

Sample preparation	*Describe the sample preparation, detailing the biological source of the cells and any tissue processing steps used.*
Instrument	*Identify the instrument used for data collection, specifying make and model number.*
Software	*Describe the software used to collect and analyze the flow cytometry data. For custom code that has been deposited into a community repository, provide accession details.*
Cell population abundance	*Describe the abundance of the relevant cell populations within post-sort fractions, providing details on the purity of the samples and how it was determined.*
Gating strategy	*Describe the gating strategy used for all relevant experiments, specifying the preliminary FSC/SSC gates of the starting cell population, indicating where boundaries between "positive" and "negative" staining cell populations are defined.*

☐ Tick this box to confirm that a figure exemplifying the gating strategy is provided in the Supplementary Information.

Magnetic resonance imaging

Experimental design

Design type	Resting-state MRI with structural T1, T2, T2s-weighted, diffusion or perfusion imaging
Design specifications	*Specify the number of blocks, trials or experimental units per session and/or subject, and specify the length of each trial or block (if trials are blocked) and interval between trials.*
Behavioral performance measures	*State number and/or type of variables recorded (e.g. correct button press, response time) and what statistics were used to establish that the subjects were performing the task as expected (e.g. mean, range, and/or standard deviation across subjects).*

Acquisition

Imaging type(s)	We used structural, perfusion and diffusion imaging in our study.
Field strength	7T (preclinical)
Sequence & imaging parameters	A T1-weighted rapid acquisition with relaxation enhancement (RARE) sequence was used to obtain anatomical images (TE/TR 9/1200ms, with 70 x 70 x 500 µm3 spatial resolution). T2-weighted images were acquired using a multi-slice multi-echo (MSME) sequence (TE/TR 50/3000ms with 70 x 70 x 500 µm3 spatial resolution). 3D T2*-weighted gradient echo imaging with flow compensation (GEFC, spatial resolution of 93 x 70 x 70 µm interpolated to an isotropic resolution of 70 µm) with TE/TR 9/50 ms and a flip angle of 15° was performed to visualize PhySIOMIC.
Area of acquisition	Whole-brain scans were used in our study.

Diffusion MRI ☒ Used ☐ Not used

Parameters	Diffusion-weighted images (DWI) were acquired using a standard spin echo imaging modified with a Stejskal-Tanner gradient scheme (TE/TR 38ms/2000ms, with 75 x 75 x 500 µm3 spatial resolution, giving an in plane resolution of 100x78mm, slice thickness of 0.75mm, one direction diffusion gradient, in the frequency encoding direction) with a b factor of 1000s/mm2 and one averaging.

Preprocessing

Preprocessing software	Preprocessing was realised using Slicer software (v4.11).
Normalization	Data were normalized (for T2s-wieighted images) with N4ITK Bias Correction Filter.
Normalization template	We used a BSpline grid resolution of 1,1,1, with Number of iterations : 50,40,30, and convergence threshold set at 0.001;

BSpline order : 3; Shrink factor : 4.

Noise and artifact removal

Describe your procedure(s) for artifact and structured noise removal, specifying motion parameters, tissue signals and physiological signals (heart rate, respiration).

Volume censoring

Define your software and/or method and criteria for volume censoring, and state the extent of such censoring.

Statistical modeling & inference

Model type and settings

Specify type (mass univariate, multivariate, RSA, predictive, etc.) and describe essential details of the model at the first and second levels (e.g. fixed, random or mixed effects; drift or auto-correlation).

Effect(s) tested

Define precise effect in terms of the task or stimulus conditions instead of psychological concepts and indicate whether ANOVA or factorial designs were used.

Specify type of analysis: ☒ Whole brain ☐ ROI-based ☐ Both

Statistic type for inference

(See Eklund et al. 2016)

Specify voxel-wise or cluster-wise and report all relevant parameters for cluster-wise methods.

Correction

Describe the type of correction and how it is obtained for multiple comparisons (e.g. FWE, FDR, permutation or Monte Carlo).

Models & analysis

n/a	Involved in the study
☐	☐ Functional and/or effective connectivity
☐	☒ Graph analysis
☐	☐ Multivariate modeling or predictive analysis

Functional and/or effective connectivity

Report the measures of dependence used and the model details (e.g. Pearson correlation, partial correlation, mutual information).

Graph analysis

Report the dependent variable and connectivity measure, specifying weighted graph or binarized graph, subject- or group-level, and the global and/or node summaries used (e.g. clustering coefficient, efficiency, etc.).

Multivariate modeling and predictive analysis

Specify independent variables, features extraction and dimension reduction, model, training and evaluation metrics.
